# Human rights in sport and democratic attitudes among student-athletes: a cross-sectional survey at a Turkish university

**DOI:** 10.3389/fpsyg.2026.1760960

**Published:** 2026-02-20

**Authors:** Reşat Sadik

**Affiliations:** Department of Sports Management, Faculty of Sport Sciences, Duzce Universitesi, Düzce, Türkiye

**Keywords:** democratic attitudes, human rights in sport, rights perception, sport and democracy, sport sciences students

## Abstract

**Introduction:**

Human rights and democratic orientations are often considered mutually reinforcing. However, in institutional settings such as sport, this relationship may be shaped by governance structures and everyday experiences of authority. This study examined associations between Sport Sciences students’ perceptions of human rights in sport and their democratic attitudes at a Faculty of Sport Sciences at a Turkish university.

**Methods:**

Using a cross-sectional survey design, data were collected from 232 students (117 female, 115 male) using the Human Rights in Sport Scale (Personal, Social, Solidarity Rights) and the Democratic Attitude Scale (Respect for Diversity, Sensitive Behaviour, Glorification of Power, Social Responsibility).

**Results:**

Males scored higher than females on Personal and Social Rights, and students who did not exercise regularly reported higher Personal and Social Rights than regular exercisers. No departmental or income differences were observed. Correlations between rights perceptions and democratic attitudes were generally small and varied across subdimensions. In hierarchical regression models controlling for gender, regular exercise, department, and income, Solidarity Rights positively predicted Respect for Diversity, whereas Personal and Social Rights were negatively associated with Glorification of Power.

**Discussion:**

Overall, the findings indicate that the relationship between perceptions of human rights in sport and democratic attitudes is complex rather than uniformly positive. The results suggest that different dimensions of rights consciousness are linked to distinct orientations towards authority and diversity in sporting settings.

## Introduction

Human rights and democracy are often discussed as mutually supportive pillars of modern social life: democracy provides institutional channels for voice and participation, while human rights define the normative limits and guarantees that protect human dignity and agency (Dahl, 2003; Can, 2019). Yet the relationship between rights endorsement and democratic orientations may be neither linear nor uniformly positive across contexts (Jost and Banaji, 1994; Fraser, 2014). In practice, attitudes towards rights can be shaped by perceptions of the accessibility of institutional remedies, proximity to decision-making structures, and the everyday legitimacy of authority within a given social setting (Dworkin, 2002; Giulianotti and McArdle, 2014). These considerations are particularly relevant in sport, where rules, hierarchies, selection processes, and surveillance coexist with ideals of fairness and inclusion.

A normative foundation helps explain why rights matter in sport as a social institution. From Heraclitus to Aristotle, logos—the capacity for reason and speech—has been treated as a defining human trait and a basis for moral agency (Rasmussen and Uyl, 1991). In that tradition, human flourishing depends on virtue and right action (Ağaoğulları, 1988), and rights safeguard the realm of action necessary for such flourishing. Rand (1999) grounds rights in the moral value of human life, while Donnelly (1995) emphasises their universal and inviolable nature across persons and contexts. Kuçuradi (2007) further distinguishes rights by their bearers (individual versus group) and by type, differentiating fundamental personal inviolabilities from civil rights. Mathieu (1994) shows how international treaties and their domestic adoption render rights enforceable. Taken together, these perspectives support a rights-based approach to evaluating social institutions—including sport—by assessing whether institutional.

From a sports sociology perspective, sport is an institutional field shaped by governance arrangements, power relations, and normative expectations that structure participation, recognition, and compliance (Donnelly, 2008; Giulianotti and McArdle, 2014). These institutional features make sport a revealing context for examining how rights are understood and enacted in everyday practice. Sport is a social institution with governance structures, power relations, and norms that can uphold or violate rights. Within international frameworks, one strand of the rights discourse emphasises the right to participate in sport. The International Olympic Committee states that “the practice of sport is a human right” (International Olympic Committee, 2014), echoing Coubertin’s inclusive ideal that “sport is for all.” Similarly, the Council of Europe affirmed in 1976 that every individual has the right to participate in sport, framing participation as a fundamental entitlement (Donnelly, 2008). This participation-oriented strand is important because it positions sport as an arena of access, equality, and social inclusion (Sadık, 2014; Kartal, 2020).

A second strand focuses on human rights in sport, that is, the concrete protections, entitlements, and obligations that should govern sport environments once participation occurs. From this perspective, sport settings should be safe, respectful, and accountable, and athletes’ experiences provide a direct lens for evaluating institutional rights protections (World Players Association, 2017; Schwab, 2017, 2018). Importantly, these two strands—the right to sport and rights in sport—are related yet analytically distinct. Confusing them can obscure whether the key question concerns access to sport or the quality and justice of sport environments. In the present study, the primary focus is rights in sport as perceived within sport settings, while recognising that participation rights form part of the broader normative backdrop.

Despite strong normative commitments, a documented gap remains between rights language and rights implementation in sport. Athletes often report limited awareness of their rights or uncertainty about how to exercise them in practice: Tuakli-Wosornu et al. (2022) report knowledge gaps and show that a substantial proportion of athletes did not know their rights in sport contexts. Practice-level risks are also well documented, including privacy and data-protection issues related to doping controls and medical information (Schneider, 2014), as well as dissatisfaction with insufficient safeguards (Patel, 2021). These patterns suggest that declarative recognition of rights may outpace practical delivery mechanisms, such as education, reporting channels, and effective remedies. Prior work in sport governance highlights a persistent discourse–practice gap, whereby rights are widely affirmed in normative documents, yet effective enforcement, independent oversight, and accessible remedies remain limited in everyday sport settings (Donnelly, 2008; Sadık, 2014; Giulianotti and McArdle, 2014; Schwab, 2017, 2018).

Democracy, meanwhile, signifies not only popular sovereignty but also a lived civic ethos grounded in pluralism, responsibility, tolerance, and the protection of rights and freedoms (Dahl, 2003; Baysal, 1981; Demiröz, 2010). Democratic attitudes—such as respect for diversity and social responsibility—shape everyday behaviour and civic engagement. Sport environments are prominent sites where such attitudes can be cultivated and demonstrated by participants, officials, and supporters. In educational sport settings, the relationship between rights awareness and democratic orientations may shape how individuals handle authority, manage conflict, recognise difference, and include others.

However, greater endorsement of rights does not necessarily translate into uniformly “higher” democratic attitudes across all dimensions. Rights endorsement can coexist with diverse understandings of authority, hierarchy, and pluralism. This is where a more explicit theoretical lens becomes necessary. First, organisational socialisation and legitimacy processes may shape whether individuals interpret rights as practically attainable or as aspirational ideals. System-justification theory, for example, suggests that individuals may come to view existing institutional arrangements as legitimate, even when these arrangements constrain rights-claiming (Jost and Banaji, 1994). Second, capabilities and justice perspectives emphasise that rights are not only declarations but also depend on material and procedural conditions that enable people to convert formal entitlements into real opportunities (Nussbaum, 1999; Fraser, 2014; Dworkin, 2002). Thus, rights perceptions may reflect perceived efficacy, voice, and access to resources, rather than purely abstract moral commitment. In this sense, the rights–democracy link in sport is theoretically plausible but can be ambivalent and multidirectional, depending on personal experience and structural factors.

Prior research documents gaps in rights awareness and implementation in sport (Schneider, 2014; Patel, 2021; Tuakli-Wosornu et al., 2022), yet it has less frequently examined how perceptions of rights in sport relate to broader democratic orientations within the same individuals. Against this backdrop, the present study investigates associations between perceptions of human rights in sport and democratic attitudes among Sport Sciences students in the national context of Türkiye. The study is positioned at the intersection of social psychology, sport sciences, and legal sociology, treating rights perceptions as a dimension of legal consciousness within sport environments and democratic attitudes as civic orientations relevant to pluralism and participation.

In line with the above framework, a uniformly positive relationship between rights endorsement and democratic attitudes is not necessarily assumed. Instead, the relationship is treated as potentially complex—coexisting with diverse orientations towards authority and diversity—and shaped by personal and structural factors, including gender and participation status. Accordingly, the study addresses two objectives: (i) to examine whether the three subdimensions of the Human Rights in Sport Scale (Personal, Social, Solidarity) vary across demographic groups (gender, regular physical activity, department, and income), and (ii) to test the zero-order correlations between these rights subdimensions and the four factors of the Democratic Attitude Scale (Respect for Diversity, Sensitive Behaviour, Glorification of Power, Social Responsibility). By combining group comparisons with correlation analysis, the study aims to identify the directions and strengths of these relationships—highlighting where rights perceptions and democratic attitudes align and where they diverge. This evidence can inform athlete-centred rights education and participatory programmes in sport environments by indicating which democratic dimensions may require explicit educational reinforcement alongside rights literacy.

## Methods

### Research design

A quantitative relational screening (cross-sectional correlational) design was utilised. This design examines the presence and strength of associations among variables based on numerical data and is commonly used to address questions such as “to what extent” and “how strongly” variables relate to one another (Büyüköztürk et al., 2017).

### Study group

The sample comprised 232 students (117 female, 115 male) enrolled in the Faculty of Sport Sciences in the spring semester of the 2024–2025 academic year. In this institutional context, admission to the Faculty requires sport-related qualifications and/or prior sport participation; therefore, participants are described as “student-athletes” based on their athletic background at programme entry. However, current regular exercise was treated as a measured characteristic rather than an inclusion criterion.

To improve representativeness within the accessible faculty population and reduce selection bias, intact classes (clusters) were randomly selected, and all eligible students in those classes were invited to participate. This cluster-based approach is commonly recommended in educational and social research when sampling from naturally occurring groups such as classes (Özdamar, 2003).

### Data collection instruments

Data were collected using the following instruments:

Demographic questionnaire. A researcher-developed form was used to collect information on gender, regular physical activity status (yes/no), department (Physical Education Teaching, Sports Management, Coaching Education), and self-reported income level (low/moderate/high). Regular physical activity was operationalised as a dichotomous self-report indicator (yes/no).

Human Rights in Sport Scale (SİHTÖ; Sadık, 2014). The SİHTÖ is a 29-item measure comprising three subdimensions: Personal Rights (12 items), Social Rights (9 items), and Solidarity Rights (8 items). *Items are rated on a 5-point Likert scale (1 = strongly disagree to 5 = strongly agree).* Possible score ranges are 29–145 for the total scale; 12–60 for Personal Rights; 9–45 for Social Rights; and 8–40 for Solidarity Rights. In the original validation study, internal consistency coefficients were reported as *α* = 0.88 (Personal), α = 0.79 (Social), α = 0.83 (Solidarity), and α = 0.82 (Total). In the present sample, the total scale reliability was α = 0.87.

Democratic Attitude Scale (DTÖ; Sincar et al., 2019). The DTÖ is a 16-item, four-factor instrument assessing Respect for Diversity, Sensitive Behaviour, Glorification of Power, and Social Responsibility. Items are rated on a 5-point Likert scale (1 = strongly disagree to 5 = strongly agree). The developers reported acceptable model fit indices (χ^2^/df = 1.809, GFI = 0.936, AGFI = 0.910, NNFI/TLI = 0.891, CFI = 0.912, RMSEA = 0.05, RMR = 0.043) and internal consistency (α = 0.748). In the present sample, the total scale reliability was α = 0.85.

Higher SİHTÖ scores indicate stronger endorsement of human rights in sport across the personal, social, and solidarity domains. For the DTÖ, higher scores on Respect for Diversity, Sensitive Behaviour, and Social Responsibility reflect more democratic orientations, whereas higher scores on Glorification of Power indicate stronger legitimisation of hierarchical/authoritarian power. The full item wordings for both scales are provided in Supplementary material.

### Procedure (data collection)

Data were collected in classroom settings during scheduled class times in the Faculty of Sport Sciences. After a brief explanation of the study’s purpose and ethical assurances (voluntary participation, anonymity, confidentiality), paper-based questionnaires were distributed to students and completed in a single session. No identifying information was collected. Completed forms were collected immediately by the researcher/instructor team. Students who did not wish to participate were free to decline without any consequences. Because data were collected in classroom settings, we aimed to reduce social desirability pressures by ensuring anonymity and emphasising that participation would not affect grades or standing.

### Data analysis

All analyses were conducted using IBM SPSS Statistics. Before inferential testing, distributional assumptions were assessed using skewness–kurtosis coefficients and Q–Q plots, and homogeneity of variances was evaluated with Levene’s test. Based on these diagnostics, parametric procedures were considered appropriate. Accordingly, independent-samples t-tests were used for two-group comparisons (e.g., gender; regular physical activity), and one-way ANOVAs were employed for comparisons involving three or more groups (e.g., department; income). When omnibus ANOVAs were significant, Tukey’s HSD *post-hoc* tests were applied if the homogeneity of variances assumption was met; otherwise, Games–Howell tests were used. Associations among continuous variables were examined using Pearson product–moment correlations. All statistical tests were two-tailed, with significance set at *α* = 0.05. Missing data were handled using listwise deletion on an analysis-by-analysis basis.

To extend the bivariate analyses and account for potential confounding, a series of hierarchical multiple regression analyses was conducted. In Step 1, gender, regular exercise status, department, and income were entered as control variables. In Step 2, the three subdimensions of the Human Rights in Sport Scale (Personal, Social, and Solidarity Rights) were added as predictors. Separate regression models were estimated for each subdimension of the Democratic Attitude Scale (Respect for Diversity, Sensitive Behaviour, Glorification of Power, and Social Responsibility) to assess the unique contribution of sport-related human rights perceptions beyond demographic and activity-related factors. Regression diagnostics indicated no problematic multicollinearity (VIFs within commonly accepted thresholds).

### Findings

This section presents the results of the statistical analyses conducted to address the research objectives. The sample’s demographic characteristics are presented first, followed by group comparisons and correlational findings.

Table 1 summarises the composition of the sample in terms of gender, regular exercise status, department, and income level.

**Table 1:** Demographic characteristics of the sample (*N* = 232).

Variable	Category	*N*	%
Gender	Female	117	50.4
	Male	115	49.6
Exercises regularly	Yes	154	66.4
	No	78	33.6
Department	Physical education teaching	66	28.4
	Sports management	82	35.3
	Coaching education	84	36.2
Income level	Low	71	30.6
	Moderate	65	28.0
	High	96	41.4
Total		232	

Table 2 summarises gender differences in SİHTÖ subscales. Males reported higher Personal and Social Rights; Solidarity Rights did not differ by gender.

**Table 2:** Gender differences in human rights in Sport (SİHTÖ) subscales (independent-samples t-tests).

Sub-dimensions	Gender	*n*	x̅	s	*t*	*p*
Personal rights	Female	117	4.4124	0.57858	3.392	0.001*
	Male	115	4.6920	0.50269		
Social rights	Female	117	3.9563	0.84039	4.462	0.001*
	Male	115	4.4502	0.79333		
Solidarity rights	Female	117	3.4626	0.59117	0.567	0.556
	Male	115	3.5025	0.42485		
Total		232				

*p < 0.05.

Table 3 shows that students who did not exercise regularly reported higher Personal and Social Rights than regular exercisers, whereas Solidarity Rights did not differ by activity status.

**Table 3:** Differences in human rights in sport (SİHTÖ) subscales by regular exercise status (independent-samples t-tests).

Sub-dimensions	Regular Exercise	*n*	x̅	s	*t*	*p*
Personal rights	No	78	4.7564	0.44091	4.119	0.001*
	Yes	154	4.4470	0.58437		
Social rights	No	78	4.5527	0.78546	4.168	0.001*
	Yes	154	4.0231	0.83154		
Solidarity rights	No	78	3.4860	0.41261	0.077	0.939
	Yes	154	3.4805	0.56068		
Total		232				

*p < 0.05.

As shown in Table 4, departmental affiliation was not associated with significant differences across any SİHTÖ subscale.

**Table 4:** One-way ANOVA results for SİHTÖ subscales by department.

Sub-dimensions	Variable	*n*	x̄	s	f	df	*p*
Personal rights	Physical education teaching	66	4.5202	0.69830	1.858	**2**	0.157
	Sports management	82	4.6443	0.47630			
	Coaching education	84	4.4841	0.50242			
Social rights	Physical education teaching	66	4.2054	1.00185	0.292	2	0.757
	Sports management	82	4.2507	0.81627			
	Coaching education	84	4.1495	0.76193			
Solidarity rights	Physical education teaching	66	3.4337	0.48448	0.533	2	0.588
	Sports management	82	3.5218	0.47840			
	Coaching education	84	3.4821	0.57172			
	Total	232					

Bold values should indicate statistically significant results only (*p* < 0.05).

Table 5 shows that income level did not significantly differentiate scores across any SİHTÖ subscale.

**Table 5:** One-way ANOVA results for SİHTÖ subscales by income level.

Sub-dimensions	Variable	*n*	x̄	s	f	df	*p*
Personal rights	Low	71	4.3102	0.59631	1.759	2	0.147
	Moderate	65	4.4441	0.57530			
	High	96	4.3841	0.49242			
Social rights	Low	71	4.2244	0.50185	0.281	2	0.698
	Moderate	65	4.1997	0.61827			
	High	96	4.1998	0.66103			
Solidarity rights	Low	71	3.4445	0.47449	0.511	2	0.554
	Moderate	65	3.4657	0.46820			
	High	96	3.4332	0.55173			
	Total	232					

As shown in Table 6, no significant gender differences were observed across any DTÖ subscale.

**Table 6:** Gender differences in democratic attitude scale (DTÖ) subscales (independent-samples t-tests).

Sub-dimensions	Gender	*n*	x̄	s	*t*	*p*
Respect for diversity	Female	117	3.3675	0.76742	0.39	0.70
	Male	115	3.1948	0.77616		
Sensitive behaviour	Female	117	3.6132	0.74604	0.229	0.82
	Male	115	3.4304	0.78678		
Glorification of power	Female	117	1.9808	0.91217	0.685	0.51
	Male	115	1.7268	1.08701		
Social responsibility	Female	117	4.0627	0.85313	1.163	0.25
	Male	115	3.8957	0.94008		
Total		232				

Table 7 shows that regular exercise participation was not significantly associated with any DTÖ subscale.

**Table 7:** Differences in democratic attitude (DTÖ) subscales by regular exercise status.

Sub-dimensions	Variable	*n*	x̄	s	*t*	*p*
Respect for diversity	No	78	3.1615	0.72725	0.334	0.74
	Yes	154	3.3429	0.79334		
Sensitive behaviour	No	78	3.4936	0.73522	0.135	0.89
	Yes	154	3.5373	0.78942		
Glorification of power	No	78	1.7318	1.24473	0.109	0.91
	Yes	154	1.9172	0.86274		
Social responsibility	No	78	4.0129	0.84583	0.132	0.90
	Yes	154	3.9784	0.92780		
Total		232				

As shown in Table 8, DTÖ subscale scores did not differ significantly by department (all *p* > 0.05).

**Table 8:** One-way ANOVA results for DTÖ subscales by department.

Sub-dimensions	Variable	*n*	x̄	s	f	df	*p*
Respect for diversity	Physical education teaching	66	3.3061	0.68408	2.472	2	0.087
	Sports management	82	3.1390	0.73413			
	Coaching education	84	3.4024	0.86233			
Sensitive behaviour	Physical education teaching	66	3.6364	0.68245	2.469	2	0.086
	Sports management	82	3.3750	0.75793			
	Coaching education	84	3.5774	0.83140			
Glorification of power	Physical education teaching	66	1.9306	1.36074	0.680	2	0.508
	Sports management	82	1.7520	0.77035			
	Coaching education	84	1.8958	0.88598			
Social responsibility	Physical education teaching	66	4.0909	0.80500	2.135	2	0.121
	Sports management	82	3.8171	0.92244			
	Coaching education	84	4.0516	0.93353			
Total		232					

Table 9 shows that income level was not significantly associated with differences across any DTÖ subscale.

**Table 9:** One-way ANOVA results for DTÖ subscales by income level.

Sub-dimensions	Variable	*n*	x̄	s	f	df	*p*
Respect for diversity	Low	71	3.3634	0.64081	0.571	2	0.566
	Moderate	65	3.2369	0.76067			
	High	96	3.2521	0.87214			
Sensitive behaviour	Low	71	3.6585	0.66580	2.034	2	0.133
	Moderate	65	3.5308	0.73886			
	High	96	3.4167	0.85044			
Glorification of power	Low	71	1.8920	0.79973	1.097	2	0.336
	Moderate	65	1.9769	1.41056			
	High	96	1.7448	0.79883			
Social responsibility	Low	71	4.1643	0.82975	2.003	2	0.087
	Moderate	65	3.9641	0.84804			
	High	96	3.8542	0.96586			
Total		232					

As shown in Table 10, SİHTÖ subscales were positively intercorrelated (*r* = 0.256–0.692, *p* < 0.001). Cross-construct associations were generally small. Personal and Social Rights were negatively associated with Respect for Diversity and Glorification of Power, whereas Solidarity Rights showed positive associations with both dimensions. Sensitive Behaviour was weakly negatively related to Social Rights, while Social Responsibility was not significantly associated with any SİHTÖ subscale.

**Table 10:** Pearson correlations between human rights in sport (SİHTÖ) and democratic attitudes (DTÖ) subscales.

		Personal rights	Social rights	Solidarity rights	Respect for diversity	Sensitive behaviour	Glorification of power	Social responsibility
Personal rights	Pearson correlation	1						
	*p*							
	*N*	232						
Social rights	Pearson correlation	0.692^**^	1					
	*p*	0.001						
	*N*	232	232					
Solidarity rights	Pearson correlation	0.375^**^	0.256^**^	1				
	*p*	0.001	0.001					
	*N*	232	232	232				
Respect for diversity	Pearson correlation	−0.155^*^	−0.188^**^	0.167^*^	1			
	*p*	0.018	0.004	0.011				
	*N*	232	232	232	232			
Sensitive behaviour	Pearson correlation	−0.112	−0.142^*^	0.059	0.906^**^	1		
	*p*	0.089	0.030	0.369	0.001			
	*N*	232	232	232	232	232		
Glorification of power	Pearson correlation	−0.295^**^	−0.298^**^	0.161^*^	0.382^**^	0.314^**^	1	
	*p*	0.001	0.001	0.014	0.001	0.001		
	*N*	232	232	232	232	232	232	
Social responsibility	Pearson correlation	−0.041	−0.070	−0.011	0.735^**^	0.894^**^	0.048	1
	*p*	0.534	0.290	0.873	0.001	0.001	0.462	

**Correlation is significant at the 0.01 level (2-tailed). *Correlation is significant at the 0.05 level (2-tailed).

To examine whether the observed associations persisted after accounting for demographic differences, we estimated hierarchical regression models with each Democratic Attitude subscale as the dependent variable.

As shown in Table 11, adding the Human Rights in Sport subscales (Step 2) meaningfully improved model fit for Respect for Diversity and Glorification of Power, whereas changes were small for Sensitive Behaviour and negligible for Social Responsibility. In Step 2, Solidarity Rights emerged as a positive predictor of Respect for Diversity, while Social Rights showed a small negative association. For Glorification of Power, Personal and Social Rights were negative predictors, whereas Solidarity Rights was positively associated with this dimension. Full regression coefficients are reported in Table 11.

**Table 11:** Hierarchical multiple regression models predicting democratic attitude subscales from human rights in sport subscales (*N* = 232).

Predictor (Step 2)	B	SE B	*β*	*p*
Panel A. Respect for diversity				
Personal rights	0.014	0.088	0.014	0.874
Social rights	−0.156	0.056	−0.220	0.006
Solidarity rights	0.350	0.071	0.351	0.001
Model fit: Step 1 R^2^ = 0.039; Step 2 R^2^ = 0.119; ΔR^2^ = 0.080				
Panel B. Sensitive behaviour				
Personal rights	0.022	0.086	0.022	0.799
Social rights	−0.104	0.055	−0.153	0.060
Solidarity rights	0.110	0.069	0.115	0.113
Model fit: Step 1 R^2^ = 0.042; Step 2 R^2^ = 0.067; ΔR^2^ = 0.024				
Panel C. Glorification of power				
Personal rights	−0.499	0.125	−0.330	0.001
Social rights	−0.236	0.079	−0.239	0.003
Solidarity rights	0.639	0.101	0.389	0.001
Model fit: Step 1 R^2^ = 0.050; Step 2 R^2^ = 0.296; ΔR^2^ = 0.246				
Panel D. Social responsibility				
Personal rights	0.057	0.103	0.048	0.579
Social rights	−0.034	0.065	−0.042	0.601
Solidarity rights	0.041	0.082	0.036	0.619
Model fit: Step 1 R^2^ = 0.042; Step 2 R^2^ = 0.046; ΔR^2^ = 0.004				

Step 1 included gender, regular exercise status, department, and income. Step 2 added Personal, Social, and Solidarity Rights (SİHTÖ). B = unstandardised coefficient; SE = standard error; β = standardised coefficient. R^2^ denotes the variance explained; ΔR^2^ denotes the change from Step 1 to Step 2. Two-tailed tests, α = 0.05.

## Discussion

This study examined how student-athletes perceive human rights in sport (Personal, Social, and Solidarity Rights) and how these perceptions relate to their democratic attitudes (Respect for Diversity, Sensitive Behaviour, Glorification of Power, and Social Responsibility). In addition to group comparisons, hierarchical regression models were employed to examine these relationships and to assess whether the associations between perceptions of human rights in sport and democratic attitudes remained significant after statistically controlling for gender, regular exercise, department, and income.

Consistent with expectations, male students reported higher endorsement of Personal and Social Rights than female students, whereas Solidarity Rights did not differ by gender. Rather than interpreting this as a straightforward “pro-rights” effect, this pattern is situated within a capability and recognition framework (Nussbaum, 1999; Fraser, 2014). Personal and Social Rights primarily reflect individual entitlements and institutional guarantees—dimensions closely linked to perceived agency, access to decision-making structures, and confidence in procedural fairness. In many sporting settings, male athletes tend to occupy more central positions within organisational hierarchies, which may enhance their perceived capacity to claim and exercise rights. From a capabilities perspective, unequal access to voice, resources, and procedural channels can produce uneven “capabilities for rights-claiming,” such that rights endorsement reflects perceived efficacy rather than purely abstract moral commitment (Dworkin, 2002).

Although some perspectives might expect stronger rights-supportive attitudes among women given their historically constrained position in sport, our findings align with mixed evidence in the literature (Sadık, 2014; Şemşek et al., 2017; Çavuşoğlu et al., 2020; Soyer et al., 2024), suggesting that gender differences in rights perceptions are context-dependent rather than uniform across settings. This underscores the importance of examining how organisational climate, coach behaviour, and access to participatory mechanisms shape gendered experiences of rights in sport. This expectation is consistent with scholarship emphasising structural gender inequalities and unequal access to resources and visibility in sport, which can shape rights consciousness and rights-claiming positions (Cooky and Messner, 2018; Lemmon, 2019; Patel, 2021).

Students who did not exercise regularly reported higher Personal and Social Rights than regular exercisers, whereas Solidarity Rights remained unchanged. Although counterintuitive at first glance, this pattern can be interpreted through organisational socialisation and system-justification processes (Jost and Banaji, 1994). Daily training routines often embed athletes within hierarchical structures characterised by selection, performance monitoring, and compliance with authority. Over time, such environments may normalise deference to coaches and institutions, reducing emphasis on individual or institutional rights. In contrast, students less immersed in these routines may retain more idealised or rights-maximalist views, relatively unconstrained by everyday organisational realities. This interpretation highlights the need to consider power distance, coach autonomy support, and institutional culture when examining rights perceptions in sport. Rather than assuming that greater exposure to sport automatically increases rights awareness, the findings suggest that participation can also reproduce hierarchical norms that temper rights endorsement.

No significant differences were observed in human rights perceptions across departments or income groups. Given that all participants were embedded within the same faculty—sharing similar curricula, institutional rules, and sporting culture—environmental homogeneity likely reduced group differences. This interpretation aligns with prior research using single-faculty samples (Sadık, 2014; Çavuşoğlu et al., 2020). Future multi-institutional studies are needed to assess whether these patterns persist across universities with differing governance structures and educational priorities.

For the Democratic Attitude Scale, no significant differences were found by gender, regular exercise, department, or income. This aligns with prior research indicating limited variation in democratic orientations within university populations (Karahan et al., 2006; Gömleksiz and Çetintaş, 2011; Yaşar Ekici, 2014). These results suggest that democratic dispositions are relatively stable across demographic subgroups in this cohort, reinforcing the analytical value of focusing on variation in rights perceptions rather than on baseline democratic attitudes.

The coexistence of gender and activity differences in human rights perceptions—alongside largely uniform democratic attitudes—mirrors mixed patterns reported in previous sport-related studies (Sadık, 2014; Şemşek et al., 2017; Turan et al., 2018; Çavuşoğlu et al., 2020; Soyer et al., 2024) and aligns with broader evidence of persistent gaps in athletes’ rights knowledge and implementation (Tuakli-Wosornu et al., 2022; Schneider, 2014; Patel, 2021). Research in educational contexts further indicates that structured rights training can enhance social-rights awareness and democratic orientations (Doğar and Çalı, 2023), supporting the conceptual linkage between human rights and democracy (Can, 2019).

On this basis, three practical implications emerge. First, sport curricula should systematically integrate rights literacy, including anti-discrimination, due process, and inclusion principles. Second, in high-intensity training environments, participatory structures such as athlete councils, confidential feedback mechanisms, and shared decision-making processes should be established to strengthen awareness of Personal and Social Rights—particularly among women and regular exercisers. Third, sport organisations should adopt measurement-based governance by regularly monitoring athletes’ confidence in reporting violations and perceptions of inclusion, and by aligning corrective actions with athlete-centred protection systems.

Bivariate correlations revealed generally small yet theoretically meaningful associations between human rights perceptions and democratic attitudes. Personal and Social Rights were negatively associated with Respect for Diversity and Glorification of Power, whereas Solidarity Rights were positively associated with both dimensions. These mixed patterns reinforce our central argument that the relationship between human rights perceptions and democratic attitudes in sport is neither linear nor uniformly positive.

To move beyond simple correlations, hierarchical regression analyses were conducted (Table 11), controlling for gender, regular exercise, department, and income. Adding the Human Rights in Sport subscales (Step 2) increased explained variance for Respect for Diversity (ΔR^2^ = 0.080) and Glorification of Power (ΔR^2^ = 0.246), but only modestly for Sensitive Behaviour (ΔR^2^ = 0.024) and not for Social Responsibility (ΔR^2^ = 0.004). After accounting for controls, Solidarity Rights positively predicted Respect for Diversity, suggesting that a collective-oriented rights framing may foster appreciation of pluralism. Notably, after controls, Social Rights showed a small negative association with Respect for Diversity. This pattern may reflect that, in hierarchical sport settings, a stronger emphasis on institutional/organisational guarantees can be interpreted in procedural terms (e.g., rights within existing authority structures) rather than as broader commitments to difference and inclusion. Given the cross-sectional design and the small effect size, this interpretation should be treated as tentative.

Taken together, these findings indicate two partially distinct pathways linking rights perceptions to democratic attitudes. An individual-entitlement orientation (Personal and Social Rights) aligns with resistance to hierarchical power but may coexist with weaker endorsement of diversity. In contrast, a solidarity-oriented rights perspective appears compatible with both respect for diversity and acceptance of legitimate authority, suggesting a form of paternalistic collectivism (Fiske, 2018). Practically, this implies that rights education alone may unintentionally reinforce a benevolent hierarchy unless accompanied by explicit pedagogy that promotes pluralism, critical reflection, and participatory governance.

Several limitations should be considered when interpreting the results. First, the cross-sectional design precludes causal inference; longitudinal or experimental approaches are needed to clarify the direction of the relationship between rights perceptions and democratic orientations. Second, reliance on self-report measures may introduce social desirability bias, a common challenge in institutional settings characterised by formal hierarchies. Furthermore, current exercise status was measured as a dichotomous variable, which does not capture variations in training intensity or competitive level (e.g., elite vs. recreational). Future research should employ multi-site samples and mixed-methods approaches—including qualitative interviews—to capture the nuanced ways in which athletes negotiate rights and authority in practice. Additionally, examining mediating mechanisms such as power distance and organisational justice would further enrich understanding of these complex relationships.

## Conclusion

This study shows that student-athletes’ perceptions of human rights in sport vary systematically by gender and level of sport participation: males and non-exercisers reported higher Personal and Social Rights, whereas Solidarity Rights remained stable across groups. Department and income had no significant effect on rights perceptions. Democratic attitudes, by contrast, showed no meaningful variation across demographic or activity-related categories, suggesting a relatively homogeneous baseline of civic orientations within this cohort.

Both correlation and regression analyses show that the relationship between human rights perceptions and democratic attitudes is complex rather than uniformly positive. Individual-oriented rights (Personal and Social) tend to align with lower endorsement of hierarchical power but are not consistently linked to stronger respect for diversity. Solidarity-oriented rights, however, relate positively to both diversity and legitimate authority, indicating multiple coexisting pathways linking rights, pluralism, and governance preferences.

Rather than assuming a simple “more rights equals more democracy” relationship, our findings highlight the need for integrated educational approaches in sport. Rights literacy should be paired with pluralism-focused pedagogy and participatory governance structures that foster dialogue, shared decision-making, and critical reflection on power relations. Ultimately, multi-institutional, mixed-methods research is required to refine our understanding of how rights consciousness and democratic attitudes interact across diverse sporting and educational contexts, informing policies that promote equity, voice, and protection in sport.

## Data Availability

The original contributions presented in the study are included in the article/Supplementary material, further inquiries can be directed to the corresponding author.
