# Supplementary material for: Human rights in sport and democratic attitudes among student-athletes: a cross-sectional survey at a Turkish university
Source: Front Psychol. 2026 Feb 20;17:1760960. doi: 10.3389/fpsyg.2026.1760960 (PMC12963339; doi:10.3389/fpsyg.2026.1760960)
Supplement: Supplementary file 1 [file Data_Sheet_1.PDF]

| n | **SİHTÖ**<br><br>Human Rights in Sport Scale (SİHTÖ)<br><br>**Scale Overview:** "There are 29 items in this scale. The approximate completion time is 10 minutes. After reading each statement, please mark the option that best reflects your level of agreement in the space provided. Please do not leave any items unmarked<br><br>**Tutum İfadeleri** | Strongly Agree | Agree | Neutral | Disagree | Strongly Disagree |
|---|---|---|---|---|---|---|
| 1 | I would like to have knowledge about human rights in sport. | | | | | |
| 2 | I feel ashamed to associate any form of harassment with sport. | | | | | |
| 3 | I appreciate having equal opportunities in sports participation. | | | | | |
| 4 | I am disturbed by coaches who use violence against athletes. | | | | | |
| 5 | I believe that sport teaches respect for others | | | | | |
| 6 | I believe that people with disabilities can participate in social life through sport. | | | | | |
| 7 | I believe that children and youth can be protected from negative influences through sport. | | | | | |
| 8 | I think that future anxiety (career uncertainty) hinders being an athlete. | | | | | |
| 9 | I want the private lives of popular athletes to be known. | | | | | |
| 10 | I want appropriate environments to be provided for athletes' right to practice their religion. | | | | | |
| 11 | I want sport education to be provided only by qualified professionals | | | | | |
| 12 | I consider offensive chanting to be a violation of personal rights. | | | | | |
| 13 | I am satisfied with the practices regarding the social security of athletes | | | | | |
| 14 | I believe that participating in sports activities is a fundamental right. | | | | | |
| 15 | I think that fairness is observed in athlete contracts. | | | | | |
| 16 | I do not approve of training methods that may cause health problems. | | | | | |
| 17 | I believe that fairness is maintained in athlete selection processes. | | | | | |
| 18 | I believe that children are trained by people with specialized sport education. | | | | | |
| 19 | I think that necessary importance is given to athlete health. | | | | | |
| 20 | I believe that recruitment in sports institutions is based on merit and fairness. | | | | | |
| 21 | I find the sports investments of local governments across different branches to be fair. | | | | | |
| 22 | I believe that sport is a part of universal culture | | | | | |
| 23 | I believe that revenues generated from sports are not utilized for social | | | | | |

| | | | | | | |
|---|---|---|---|---|---|---|
| | solidarity. | | | | | |
| 24 | I believe that sport is an activity that contributes to world peace. | | | | | |
| 25 | I believe that sport is enriched by cultural differences. | | | | | |
| 26 | I find the legal regulations regarding athletes' rights to be insufficient | | | | | |
| 27 | I think that sport contributes to social peace. | | | | | |
| 28 | I believe that discrimination occurs in the recruitment processes of sports institutions. | | | | | |
| 29 | I believe that the decisions made by sports courts are not fair | | | | | |

| n | **DTÖ**<br>**Scale Overview:** There are 16 items in this scale. The approximate completion time is 5 minutes. After reading each statement, please mark the option that best reflects your level of agreement in the space provided. Please do not leave any items unmarked<br>**Tutum İfadeleri** | Strongly Agree | Agree | Neutral | Disagree | Strongly Disagree |
|---|---|---|---|---|---|---|
| 1 | I show respect for different cultural identities. | | | | | |
| 2 | I show tolerance toward different religious beliefs. | | | | | |
| 3 | I show tolerance toward ethnic differences. | | | | | |
| 4 | I criticize my own ideology (system of thought) when necessary. | | | | | |
| 5 | I show tolerance toward different styles of dress. | | | | | |
| 6 | I have respect for labor and effort. | | | | | |
| 7 | I adhere to the rules of etiquette (courtesy) in human relations. | | | | | |
| 8 | I take responsibility for my actions. | | | | | |
| 9 | I am sensitive to social issues. | | | | | |
| 10 | I give preferential treatment to those who share my own ideology (belief, thought) | | | | | |
| 11 | I prefer to take the side of the powerful. | | | | | |
| 12 | In conflicts, I choose the side that will not harm me. | | | | | |
| 13 | I do not show compassion toward people I know to be atheists | | | | | |
| 14 | I prioritize social benefit over personal gain. | | | | | |
| 15 | I view my work as a social responsibility. | | | | | |
| 16 | I maintain a balance between individual and social values in my | | | | | |

| | | | | | | |
|---|---|---|---|---|---|---|
| | relationships. | | | | | |
